# Metacarpal Stress Fracture Is Not an Uncommon Condition in Adolescent Racket Athletes

**DOI:** 10.1155/2020/5840925

**Published:** 2020-02-08

**Authors:** Karen Nishikawa, Yuka Kimura, Daisuke Chiba, Norihiro Sasaki, Shizuka Sasaki, Shinji Nishikawa, Yasuyuki Ishibashi

**Affiliations:** ^1^Hirosaki University School of Medicine, 5 Zaifu-cho, Hirosaki, Aomori, Japan 036-8562; ^2^Department of Orthopaedic Surgery, Hirosaki University Graduate School of Medicine, 5 Zaifu-cho, Hirosaki, Aomori, Japan 036-8562; ^3^Nishikawa Hand Surgery Orthopaedic Clinic, 2-2-12 Waseda, Hirosaki, Aomori, Japan

## Abstract

**Background:**

Stress fractures of the metacarpal bones are considered uncommon. We report on 11 adolescent athletes with these stress fractures, successfully treated with cessation of sports activities. *Representative case presentation*. In case 1, a 15-year-old male tennis player presented with right hand pain of 4-week duration without an acute trauma history. Tenderness existed on palpation along the dorsal and proximal second metacarpal bone. Plain radiographs demonstrated a periosteal reaction on the proximal shaft of the second metacarpal. Racket swinging was suspended. He returned to competitive tennis 2 months after the initial visit and continues to participate without symptoms. In case 2, a 16-year-old male boxer presented with right hand pain of 2-week duration that arose while punching. Acute trauma history was absent. Tenderness existed on palpation over the third metacarpal of the right hand. Plain radiographs demonstrated no periosteal reaction or fracture line. MRI showed a high signal on the third metatarsal bone on fat suppression and a low signal on T2-weighted images. Nonoperative treatment was initiated without external fixation, and punching was suspended. He returned to boxing 1 month after the initial visit without symptoms.

**Conclusions:**

The current case series of metacarpal stress fractures demonstrate that this condition is not as rare as previously reported. Metacarpal stress fractures are generally ignored since the clinical and radiological findings are mostly unclear. If an athlete experiences hand pain without acute onset during sports activities, especially in racket sports, the presence of a metacarpal stress fracture should be assessed by MRI.

## 1. Background

Stress fractures are common overuse injuries in athletes and generally occur in the lumbar spine and lower extremities; those affecting the upper limbs are uncommon, accounting for 2.8-7.6% of all stress fractures [1, 2]. Because the number of sports-specific movements involving repetitive use of an upper limb has increased in sports such as racket sports, throwing sports, and boxing, the incidence of stress fractures in the upper limbs also may increase. Nevertheless, stress fractures of the metacarpal bones are considered relatively uncommon. We report on 11 adolescent athletes aged 13 to 24 years old with stress fractures of the metacarpal bones, successfully treated with cessation of sports activities.

## 2. Case Presentation

### 2.1. Case Report 1

**Figure 1:**
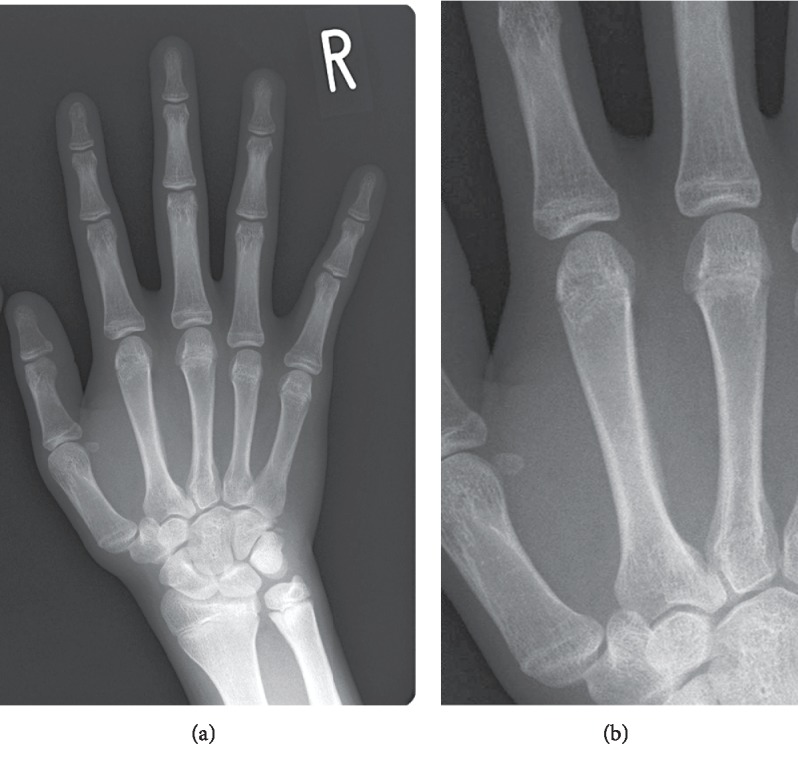
Right hand radiograph showing a periosteal reaction at the ulnar side in the shaft of the second metacarpal.

A 15-year-old male tennis player presented to our institution with right hand pain of 4-week duration without an acute trauma history. His involved side was the racket-hand side. Physical examination revealed tenderness on palpation along the dorsal and proximal second metacarpal bone. No swelling, ecchymosis, mass, or deformity was seen in the hand. He had full range of motion of the wrist and fingers in all planes without pain. Hand anteroposterior (A-P) radiographs demonstrated a periosteal reaction on the ulnar aspect of the proximal shaft of the second metacarpal (Figure 1). Second metacarpal stress fracture was diagnosed. Nonoperative treatment was initiated without external fixation. Racket swinging was suspended. He returned to competitive tennis 3 weeks after the initial visit and continues to participate without symptoms.

### 2.2. Case Report 2

**Figure 2:**
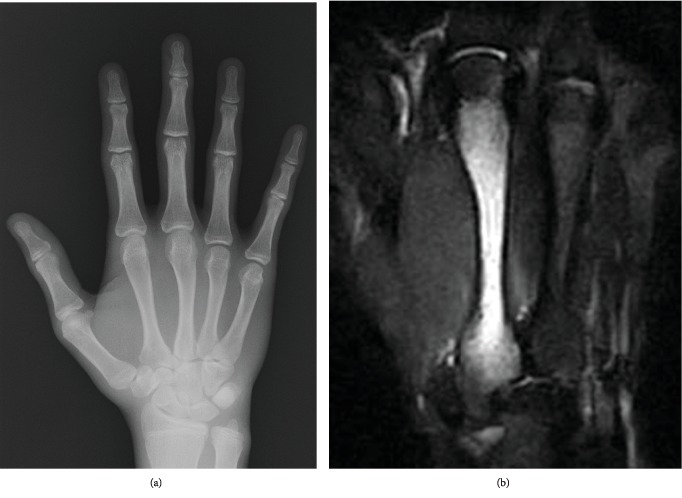
(a) Left hand radiograph showing no periosteal reaction or fracture line in the metacarpal. (b) Fat-suppressed fast spin-echo T2-weighted magnetic resonance showing high signal intensity in the shaft of the left third metacarpal.

A 16-year-old male boxer presented to our institution with right hand pain of 2-week duration that arose while punching. Acute trauma history was absent. Physical examination revealed tenderness on palpation over the third metacarpal of the right hand. Hand A-P radiographs demonstrated no periosteal reaction or fracture line (Figure 2(a)). Magnetic resonance imaging (MRI) showed a high signal on the third metatarsal bone on fat suppression and a low signal on T2-weighted images (Figure 2(b)). Third metacarpal stress fracture was diagnosed. Nonoperative treatment was initiated without external fixation, and punching was suspended. He returned to boxing 1 month after the initial visit and continues to participate without symptoms.

This case report included 11 athletes (six males and five females; mean age, 16.9 years; range, 13-24 years) diagnosed with the stress fractures of the metacarpal bones. The patient profiles and clinical results are shown in Table 1. Six patients played tennis, two played badminton, two boxed, and one bowled. Nine of the 11 patients injured the right hand and two injured the left hand. All those playing racket sports injured the dominant hand (racket-hand side). No patient had an acute trauma history. All patients except for one chronic patient had dorsal hand pain during sports activity for about 2 weeks from onset to initial visit. In ten patients, the second metacarpal bone was involved, and six patients had shaft and four had stress fractures at the base of the second metacarpal. Only one patient, who was a boxer, had a stress fracture at the shaft of the third metacarpal bone. Two of the 11 patients were diagnosed only using a plain radiograph of the hand, demonstrating periosteal or cortical stress reactions in the metacarpal bone, and nine patients underwent MRI in addition to plain radiography.

All patients were treated nonoperatively with rest and cessation of sports activity. After conservative therapy without external fixation such as a casting, the symptoms disappeared completely in all cases. Before returning to sports activity, the grip form of racket sports players was checked; if the players showed incorrect grip, their grip form was altered. In some cases, we advised to switch western grip over to eastern grip. Most patients returned to their sports activity within 6 months. All patients provided informed written consent for this study, and in particular, patients under 17 years old provided the consent with their guardian. The ethical committee of our institution approved this case report.

## 3. Discussion

Stress fractures in the metacarpal bones were previously considered to be rare; however, this report showed a relatively high number of cases. We found many metacarpal stress fractures because we could use MRI relatively easily.

The second metacarpal is the longest of all the metacarpals, with a broad base linked to the first and third metacarpals, trapezoid, trapezium, and capitate. The extensor carpi radialis longus inserts on the radial aspect of the base of the second metacarpal, and the flexor carpi radialis inserts on the anterior aspect of the base of the second metacarpal. Therefore, flexing and extending the wrist joint produce larger mechanical stress to the base of the second metacarpal and cause stress fractures distal to the insertion of these muscles.

**Figure 3:**
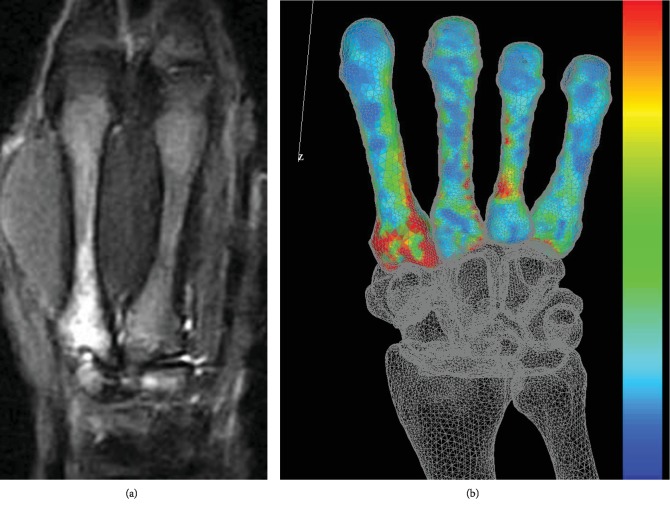
(a) Fat-suppressed T2-weighted MRI showing high signal intensity in the base of the second metacarpal. (b) Finite element analysis showing the stress distribution of the second metacarpal. Maximum stress was present at the base of the second metacarpal bone.

The second metacarpal bone bears mechanical stress at the point where players hit the ball using a racket [3]. Knudson, using a 3D motion analysis system, demonstrated increased mechanical force on the base of the second metacarpal bone when tennis players hit the ball with the forehand stroke [4]. Stress fractures of metacarpal bones typically occur at the ulnar aspect [5–8] probably because both the ligaments and the joint congruence between the second and third metacarpals are greater than those between the first and second; biomechanically, the force produced by beating the racket is directed from radial to ulnar, with the greatest tension in the medial aspect of the metacarpal base [6, 9]. Our computed tomography- (CT-) based finite element analysis (FEA) software (Mechanical Finder, Research Center for Computational Mechanics, Tokyo, Japan) showed increased mechanical stress at the base and ulnar side of the second metacarpal (Figure 3), revealing one of the causes of stress fractures of the second metacarpal bone.

**Figure 4:**
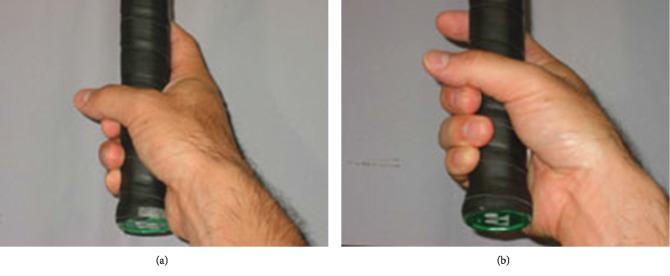
(a) Eastern and (b) western grip style.

Eight of the 11 patients played racket sports. The grip style of the racket is related to the sports-specific mechanism of stress fracture. Balius et al. reported that six of seven tennis players with stress fractures of the second metacarpal were using the semiwestern or western grip [8]. In the western grip, the palm is parallel to the surface of the racket, and hence, the wrist joint requires pronation and supination motion during racket movement, delivering mechanical stress to the second metacarpal [7]. However, in the eastern grip, the palm is perpendicular to the surface the racket (Figure 4) [8]. Waninger and Lomnardo reported that changing the grip style from western to eastern would be effective to prevent stress fractures of the metacarpal bone [9]. In some cases, western style was actually switched over to eastern style. Moreover, if the racket sports players demonstrated incorrect grip form, we tried to alter their form. In particular, the incidence of stress fracture in metacarpal bone can be likely in teenagers based on the previous [8] and current data. Some teenagers seem to be inexperienced and demonstrate incorrect form despite practicing harder. For instance, we had them grab a racket along the line connecting the metaphalangeal joint of the index finger to the hypothenar eminence. Grip size was changed to make adequate space; one-finger breath is made between the index and the thumb finger while they grab a racket. Altering incorrect grip form to correct one and grip style have potential to reduce the stress of metacarpal bone.

Two patients in this series were boxers, and one had involvement of the third metacarpal bone. Because of the characteristics of the knuckle, where the third metacarpal head protrudes the most, repetitive punching might cause stress fractures of the third metacarpal. The mechanism of metacarpal stress fractures in boxing differs from that in racket sports. Metacarpal stress fractures in other locations have been reported previously, including injuries affecting athletes in rowers with a fourth metacarpal stress fracture caused by awl gripping [10] and in a softball pitcher with a fifth metacarpal stress fracture caused by curveball pitching [11].

Stress fractures should be suspected in patients with a recent increase in physical activity or repeated excessive activities eliciting pain. There were no specific physical findings such as tenderness along the metacarpal bone in the dorsal hand or swelling. Plain radiographs usually show periosteal reaction, thickness of the cortex, and fracture lines; in early-onset cases, such abnormal signs are absent. Furthermore, these are relatively minor findings and may be easily overlooked. For early stress fracture diagnosis, MRI and bone scintigraphy are useful [12] as shown in the current case series.

Patients without apparent fracture lines in radiographs do not need external fixation, and temporarily suspending the maneuver related to the metacarpal stress fracture is sufficient. All patients in these cases returned to sports after tenderness and swelling along the metacarpal disappeared, callus on radiographs was confirmed, or bone marrow edema (high signal change) disappeared on MRI. None showed recurrence after return to sports activity.

In conclusion, we report a case series of metacarpal stress fractures and demonstrate that this condition is not as rare as previously reported. Metacarpal stress fractures are generally ignored since the clinical and radiological findings are mostly unclear. If an athlete experiences hand pain without acute onset during sports activities, especially in racket sports, the presence of a metacarpal stress fracture should be assessed by MRI.

## Figures and Tables

**Table 1:** Profile and clinical results in the eleven cases of metacarpal stress fracture.

Case	Age (years)	Sex	Time from onset to initial visit (weeks)	Metacarpal	Location	Sports	Time from initial visit to return to sports (weeks)
1	13	F	2	Second	Shaft	Badminton	4
2	14	F	1	Second	Base	Soft tennis	12
3	14	M	3	Second	Base	Badminton	4
4	15	M	4	Second	Base	Tennis	3
5	16	M	2	Third	Shaft	Boxing	4
6	16	M	2	Second	Shaft	Tennis	4
7	16	F	3	Second	Base	Tennis	10
8	18	F	1	Second	Shaft	Tennis	4
9	18	M	52	Second	Shaft	Boxing	5
10	22	F	1	Second	Shaft	Tennis	4
11	24	M	2	Second	Shaft	Bowling	4

## Abbreviations

A-P: Anteroposterior
CT: Computed tomography
FEA: Finite element analysis
MRI: Magnetic resonance imaging.
